# PASTEC: An Automatic Transposable Element Classification Tool

**DOI:** 10.1371/journal.pone.0091929

**Published:** 2014-05-02

**Authors:** Claire Hoede, Sandie Arnoux, Mark Moisset, Timothée Chaumier, Olivier Inizan, Véronique Jamilloux, Hadi Quesneville

**Affiliations:** 1 INRA, UR1164 URGI - Research Unit in Genomics-Info, Versailles, France; 2 INRA, plateforme Bio-informatique Genotoul, Mathématiques et Informatique Appliquées Toulouse UR875, Castanet-Tolosan, France; 3 INRA, LUNAM Université, Oniris, UMR1300 BioEpAR, Nantes, France; University of Poitiers, France

## Abstract

**Summary:**

The classification of transposable elements (TEs) is key step towards deciphering their potential impact on the genome. However, this process is often based on manual sequence inspection by TE experts. With the wealth of genomic sequences now available, this task requires automation, making it accessible to most scientists. We propose a new tool, PASTEC, which classifies TEs by searching for structural features and similarities. This tool outperforms currently available software for TE classification. The main innovation of PASTEC is the search for HMM profiles, which is useful for inferring the classification of unknown TE on the basis of conserved functional domains of the proteins. In addition, PASTEC is the only tool providing an exhaustive spectrum of possible classifications to the order level of the Wicker hierarchical TE classification system. It can also automatically classify other repeated elements, such as SSR (Simple Sequence Repeats), rDNA or potential repeated host genes. Finally, the output of this new tool is designed to facilitate manual curation by providing to biologists with all the evidence accumulated for each TE consensus.

**Availability:**

PASTEC is available as a REPET module or standalone software (http://urgi.versailles.inra.fr/download/repet/REPET_linux-x64-2.2.tar.gz). It requires a Unix-like system. There are two standalone versions: one of which is parallelized (requiring Sun grid Engine or Torque), and the other of which is not.

## Introduction

Transposable elements account for a high proportion of eukaryotic genomes. They are involved in a number of important processes, including genome rearrangement, heterochromatin formation and the regulation of gene expression. Wicker *et al*. [1] published a detailed classification for eukaryotic TEs. Their goals were to harmonize and clarify TE classifications and names. This classification includes both well-known classes of TEs: class I (retrotransposons) and II (DNA transposons). Five orders (LTR, DIRS, PLE, LINE, SINE) are defined for class I, and four (TIR, Crypton, Helitron, Maverick) for class II. Each order is also divided into one or several superfamilies, resulting in 29 superfamilies total. Other categories comprising non-autonomous TEs (LARD, TRIM and MITE) are considered in this classification, together with various forms of non-autonomous entities related to TEs to various degrees. This classification is based on the transposition mechanism, sequence similarities and structural relationships. Wicker *et al*. described the protein-coding domains present in each TE superfamily in particular detail.

Several computational tools have been proposed for the *ab initio* identification of repeat families (see reviews [2]; [3]). These tools generally reconstruct the consensus sequences of repeated regions. It is then useful to characterize and classify these consensus sequences, to study the TE repertoire of a genome. Two tools for the automatic classification of a large range of TEs have been proposed: TEclass [4] and REPCLASS [5]. TEclass classifies TE sequences only into two classes: Retro (class I in Wicker's classification) and DNA (class II in Wicker's classification). This tool can classify class I elements into LTRs and non-LTRs and, where possible, into SINE or LINE elements. However, TEClass cannot distinguish between the various orders of class II elements. The SVM (Support Vector Machine: supervised learning model) classifiers in this tool were trained with RepBase Update, a database of eukaryotic repetitive sequences [6], using different class lengths and vectors of tetramer and pentamer frequencies. This approach is useful when TEs show no clear similarity to known repeats.

REPCLASS can classify TEs into classes I and II, and into the orders LTR retrotransposon (grouping LTR, LARD, TRIM, DIRS and PLE from Wicker's classification), DNA transposon (grouping TIR, crypton and Polinton from Wicker's classification), LINE/SINE and helitron. In some cases, this tool can also identify the superfamily of the TE. REPCLASS consists of three modules. The first is based on homology and has the greatest impact on final decision. At this step, the software carries out a tblastx analysis against the RepBase databank. The second module (in terms of order of priority for classification) searches for structural characteristics: terminal repeats, such as LTRs and TIRs, tRNA, polyA signals and SSR. Finally, the third module searches for target site duplication (TSD) around TE copies within the genome.

Neither TEclass nor REPCLASS can classify TEs at the level of detail given by Wicker's classification (see Table 1). We therefore developed PASTEC (Pseudo Agent System for Transposable Element Classification), a modular tool able to classify TEs automatically to order level, for all nine orders of autonomous TEs defined in Wicker's classification plus three orders of non-autonomous TEs (LARD, TRIM and MITE). If PASTEC cannot classify a TE to order level, it classifies it to class level. PASTEC is also designed to filter out false-positive repeated sequences identified by *de novo* approaches. It can therefore distinguish SSRs, rDNA sequences and potential host genes from real TEs. Moreover, PASTEC can classify and recognize incomplete TEs and potentially chimeric TEs (detection of an anomaly in the evidence used to select the classification, or several classifications possible). In this last case, PASTEC chooses the best classification if it has sufficient evidence to make a decision. All evidence is written to the output file, to facilitate the manual curation of potential chimeric TEs. We tested this tool by comparing the results of these three tools on three different datasets.

**Table 1:** Comparison of the classifications obtained with the PASTEC, TEClass and RepClass tools.

PASTEC (Wicker's) Class	PASTEC (Wicker's) Order	TEClass Class	TEClass Order	RepClass Class	RepClass Order
**Class I (Retrotransposons)**					
	LTR	Retro	LTR	Class I	LTR/DIRS/PLE
	DIRS	Retro	LTR	Class I	LTR/DIRS/PLE
	PLE	Retro	LINE	Class I	LTR/DIRS/PLE
	LARD*	Retro	*na*	Class I	*na*
	TRIM*	Retro	*na*	Class I	*na*
	LINE	Retro	LINE	Class I	LINE/SINE
	SINE	Retro	SINE	Class I	LINE/SINE
**Class II (DNA transposons)**					
	TIR	DNA	DNA	Class II	TIR/Crypton/Polinton
	MITE*	DNA	DNA	Class II	TIR/Crypton/Polinton
	Crypton	DNA	*na*	Class II	TIR/Crypton/Polinton
	Helitron	DNA	*na*	Class II	Helitron
	Maverick	DNA	*na*	Class II	TIR/Crypton/Polinton

Notes: (*) Non-autonomous element. *na*: not considered by the tool.

## Materials and Methods

PASTEC was developed in the REPET package [7]. In this context, we used PASTEC to classify the consensus TE sequences found *de novo* in a genome. PASTEC uses several features of TEs to classify TE consensus sequences. It searches for structural evidence and sequence similarities stored in a MySQL database obtained during a preprocessing step. The structural features considered are TE length, presence of a LTR (long terminal repeat) or TIR (terminal inverted repeat) detected with a custom-built tool (with a minimum length of 10 bp, a minimum identity of 80%, the taking into account of reciprocal orientations of terminal repeats and a maximal length of 7000 bp), the presence of SSRs (simple sequence repeats detected with the tandem repeat finder (TRF) tool [8]), the polyA tail and an ORF (open reading frame). The blastx and tblastx routines are used to search for similarities to known TEs in Repbase Update, and the hmmer3 package [9] to search against a HMM profile databases (TE-specific or not), after translation in all six frames. Sequence similarities are also identified by blastn searches against known rDNA sequences, known host genes and known helitron ends. The databanks used are preprocessed and formatted. The Repbase Update for PASTEC can be downloaded from http://www.girinst.org/repbase/index.html, whereas the HMM profile databank formatted for PASTEC is available from the REPET download directory (http://urgi.versailles.inra.fr/download/repet/).

PASTEC classifies TEs by testing all classifications from Wicker's hierarchical TE classification system. Each possible classification is weighted according to the available evidence, with respect to the classification considered. TEs are currently classified to class and order level. PASTEC can also determine whether a TE is complete on the basis of four criteria: sequence coverage for known TEs, profile coverage, presence of terminal repeats for certain classes, presence of a polyA or SSR tail for LINEs and SINEs, and the length of the TEs with respect to expectations for the class concerned.

**Figure 1:**
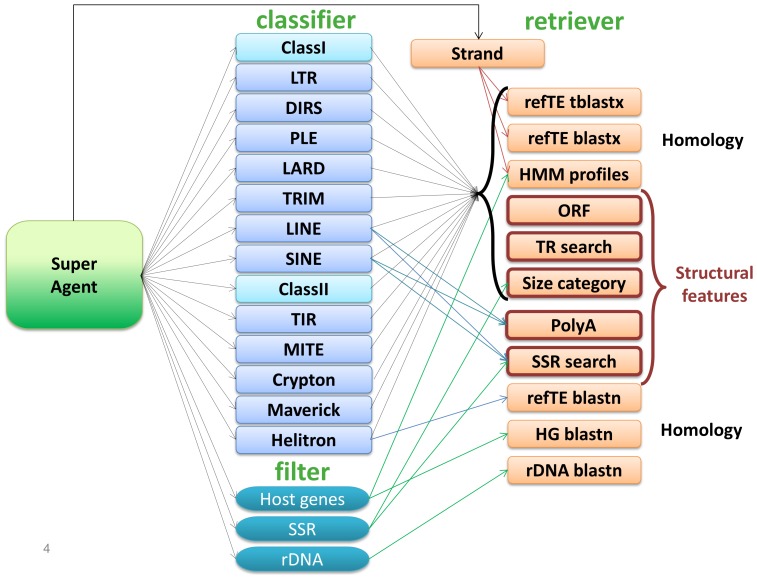
Agents implemented in the system. Orange agents are retriever agents, blue agents are classifier and filter agents. The super-agent is shown in green. The arrows indicate the principal communications between the different agents, with only requests shown.

We designed PASTEC as a modular multi-agent classifier. The system is composed of four types of agents: retrievers, classifiers, filter agents, and a super-agent (Figure 1). The retriever agents retrieve the pre-computed analysis results stored in the MySQL database. They act on the requests of the classifier or filter agents, filtering, formatting and supplying the results. The classifier and filter agents are specialized to recognize a particular category. For example, the LTR agent can determine only whether the TE is a LTR or not. The classifier and filter agents act on the request of the super-agent, deciding whether they can classify the TE or not. For example, the LTR agent decides whether the consensus TE is a LTR on the basis of the following evidence: presence of the ENV (envelope protein) profile (a condition sufficient for classification), the presence of INT (integrase), RT (reverse transcriptase), GAG (capsid protein), AP (aspartate proteinase) and RH (RNase H) profiles together with the detection of a LTR (long terminal repeat), a blast match with the sequence of a known LTR retrotransposon. The super-agent resolves classification conflicts and formats the output file. It resolves conflicts by using a confidence index normalized to 100. For example, the LTR agent calculates a confidence index with the following rules: presence of ENV profiles (+2 because this condition is sufficient for classification), presence of a long terminal repeat and an INT, GAG, RT, RH or AP profile (+1 for each profile combined with the long terminal repeat), +1 for each profile (ENV, AP, RT, RH and GAG) found in the same frame in the same ORF. If the consensus matches at least one known LTR retrotransposon, the LTR agent adds +2 for each type of blast (blastx or tblastx) at the confidence index. Finally, the length of the TE is taken into account because we add +1 if the TE without the long terminal repeat is between 4000 and 15000 bp in length, and we decrease the confidence index by 1 if the TE without the long terminal repeat is less than 1000 bp or more than 15000 bp long. The super-agent uses the maximum confidence index defined for each classifier agent to normalize the confidence index for each classification to 100 and then compare the different classifications. Advanced users can edit all decisions rules and maximum confidence indices in the Decision_rules.yaml file.

The output can be read by humans and is biologist-friendly. A single line specifies the name of the TE, its length, status, class, order, completeness, confidence index and all the features characterizing it. A status of “potential chimeric” or “OK” is assigned to the TE. If the TE is not considered to be “OK” then users must apply their own expertise. A TE is declared “potential chimeric” when at least two classifications are possible. In this case, PASTEC chooses the best status based on the available evidence, or does not classify the TE if no decision is possible. In this last case, all possible classifications are given (separated by a pipe symbol “|”). We present an example of PASTEC output in table S1. PASTEC output is a tabular file, with the columns from left to right indicating the name of the TE, its length, the orientation of the sequence, chimeric/non-chimeric status (OK indicating that the element is not potentially chimeric), class (class I in this case), order. In the first line of the example provided, the TE is a LTR. We presume that the element is complete because we have no evidence to suggest that it is incomplete, and the confidence index is 71/100. The last column summarizes all the evidence found: coding sequence evidence, such as the results of tblastX queries against the Repbase database (TE_BLRtx evidence), blastX queries against the Repbase database (TE_BLRx evidence) and profiles. A blast match is taken account if coverage exceeds 5%, and a profile is taken into account if its coverage exceeds 20% (these parameters can be edited in the configuration file). For each item of coding sequence evidence, the coverage of the subject is specified. The structural evidence is also detailed: >4000 bp indicates that TE length without terminal repeats is between 4000 and 15000 bp, the next item of information presented in the comments columns is the presence of terminal repeats: we have a LTR in this case, with an LTR length of 433 bp; two long ORFs have been identified, the last of which contains four profiles in the same frame and is up to 3000 bp long. Other evidence provided for this example includes the partial match with a *Drosophila melanogaster* gene (coverage 16.55% and the TE contains 18% SSRs). The super-agent determines whether a TE is complete based on whether it is sufficiently long, whether the expected terminal repeats or polyA tail are present, whether blast match coverage exceeds 30% and profile coverage exceeds 75%. The second line of the example corresponds to a potentially chimeric TE, for which human expertise is required.

## Results and Discussion

We compared the results of PASTEC with those of the two other classification tools, REPCLASS and TEclass, for three datasets: (i) the *A. thaliana* consensuses found in Repbase update version 15.09 (referred to hereafter as Repbase-atha), (ii) the TEs present in Repbase update 15.09 but not in Repbase 13.07 (referred to hereafter as Repbase-diff), and (iii) the whole Repbase update 15.09 from which we removed redundant TEs, i.e., those with strictly identical sequences (referred to hereafter as Repbase-all). For RepClass and PASTEC, which require a database for blast analyses, we used RepBase update 15.09 from which we removed the *A. thaliana* TEs in the first case, RepBase 13.07 in the second case and no blast database in the last case. The second case (Repbase-diff) mimics standard use: we wanted to classify TEs obtained from a new genome and, therefore, not yet known in the Repbase database. Indeed, Repbase-diff contains the TEs present in Repbase Update 15.09, but not in Repbase Update 13.07, and we provided Repbase Update 13.07 for sequence similarity searches by REPCLASS and PASTEC. So, as is usually the case in such analyses, we have unknown TEs (Repbase-diff) and a database of known TEs (Repbase Update 13.07). This made it possible to check whether the classification results for Repbase-diff were consistent with the classification given in Repbase Update 15.09. The last case is the most difficult, with attempts to classify TEs as if no known TEs are present in the database.

We compared the classification performance, in terms of the percentages of TEs well classified, misclassified relative to their annotation in the Repbase database or not classified at the level of class (Table 2) or order (Table 3), for each of the three tools. It was not possible to compare sensitivity and specificity for each order, because the three tools do not use the same classification. Nevertheless, sensitivity and specificity data are presented in the supplementary data for the orders for which this was possible. We mapped the PASTEC classification onto those used by TEclass or REPCLASS, to improve comparisons. Table 3 shows the performances obtained with PASTEC after mapping.

**Table 2:** Comparison of the performances of PASTEC, REPCLASS, and TECLASS for classification into TE classes.

Dataset (sequence ^#^)	Performance	PASTEC	REPCLASS	TECLASS
Repbase-atha (318)				
	Well classified	80.7	83.6	98.4
	Misclassified	**0.95**	3.5	1.3
	Not classified	18.4	12.9	0.3
Repbase-diff (5546)				
	Well classified	63.7	26.1	59.2
	Misclassified	**2.9**	31.8	**40.2**
	Not classified	33.4	42.1	0.5
Repbase-all (9665)				
	Well classified	52.7	20.35	53.8
	Misclassified	**5.9**	27.09	**45.8**
	Not classified	41.3	52.6	0.3

**Table 3:** Comparison of the performances of PASTEC, REPCLASS, and TECLASS for classification to TE order level.

Dataset (sequence ^#^)	Performance	PASTEC	PASTEC mapped to REPCLASS order (1)	PASTEC mapped to TECLASS order (2)	REPCLASS (3)	TECLASS (4)
Repbase-atha (318)						
	Well classified	71.4	79.7	93.7	85.5	97.5
	Misclassified	**10.2**	**1.9**	2.5	2.8	1.9
	Not classified	18.4	18.4	3.8	11.6	0.6
Repbase-diff (5546)						
	Well classified	51.3	59.1	49.7	66.9	47.7
	Misclassified	**10.9**	**3.1**	3.5	11.1	**50.3**
	Not classified	33.4	33.4	46.8	17.6	1.9
Repbase-all (9665)						
	Well classified	22.4	31.9	8.6	12.4	40
	Misclassified	**15.8**	**6.2**	6.7	33.3	**59.7**
	Not classified	61.8	61.8	84.7	50.84	0.25

Note that the classification differs between the three tools. We therefore mapped the PASTEC classification results onto those for REPCLASS (1) and TECLASS (2). (2) Mapped onto TECLASS class I orders only. (3) order considered are: DNA transposon, LTR retrotransposon, helitron, non LTR retrotransposon. (4) order considered are only LTR, LINE/SINE.

PASTEC misclassified very few TEs, regardless of the dataset tested (Table 2 and 3). These rare ambiguities mostly concerned closely related TEs: essentially MITE/TIR (427/605 −70.6% - TEs misclassified at order level in the Repbase subtraction dataset). For Repbase-diff, PASTEC was frequently more specific than the other two tools (see suppl. Data: Venn diagram and table of sensitivity and specificity of the three tools for this second dataset Tables S2, S3, S4, S5 S6, Figures S1, S2, S3, S4, S5 S6). However, it failed to classify many TEs due to a lack of useful evidence. PASTEC HMM profile detection was found to be particularly useful if the database contained no similar TE to that for which the search was carried out. For Repbase-all without blast searches, we had profile information for 3488 TEs, making it possible to classify these elements at least partially. For classification to order level, PASTEC gave better results than REPCLASS. PASTEC correctly classified more TEs than REPCLASS for the Repbase-all library (31.9% of the TEs were well classified by PASTEC on the basis of REPCLASS order, whereas REPCLASS classified only 12.4% of TEs well, and PASTEC misclassified fewer TEs for all datasets (see Table 2 and 3).

REPCLASS performed well on the Repbase-atha dataset, particularly for helitrons, thanks to its helitron_scan tool, if no TSDs were found. It was efficient, but required the genome sequence. This tool searches for 5′ and 3′ termini, including the flanking sequences of all copies found in the genome, and a subterminal hairpin-like GC-rich motif. For the other datasets, we provided no genome sequence because the TEs came from many different genomes. In such conditions, the TSD module of REPCLASS cannot be used, but good results were nevertheless obtained with this tool for the Repbase-diff dataset (67% of TEs well classified to order level, see Table 3). For this dataset, REPCLASS was more sensitive (83.6% *vs* 52.7%) but less specific (89.7% *vs* 99.9%) than PASTEC for classification to order level. By contrast, for the Repbase-all dataset (without blast homology searches), this tool gave the worst results (12.4% of TEs well classified to order level).

TEclass classified the *A. thaliana* dataset well, but despite the number of TEs correctly classified with each of the three test datasets, the percentage of TEs misclassified remained high. TEclass misclassified between 40% and 60% of TEs from the Repbase-diff and Repbase-all datasets, misclassifying more than half of the TEs at order level for these two datasets. This suggests that this tool does not recognize effectively the situations in which it cannot give a correct classification. For interpretation purposes, a lower level of misclassification with more TEs unclassified and requiring manual checking would be preferable. It should also be noted that, in this study, TEclass was at an advantage over the other tools, as we could not train TEclass with a database containing none of the TEs in the test set.

## Conclusions

In conclusion, TEclass misclassified TEs more frequently than the other tools for the two largest datasets (more than 50% of TEs for classification to order level). Moreover, the order classification of this tool is highly simplified (only four potential classifications). REPCLASS also has a simplified order classification (only four potential classifications). For these four orders, this tool performs well in conditions in which a homology search is possible (86% of Repbase-atha TEs well classified, with only 3% misclassified, 67% of Repbase-diff TEs well classified, with only 11% misclassified). However for the Repbase-all dataset, for which no homology search was possible, it performed poorly (12% of TEs well classified for 33% misclassified).

PASTEC was found to have many advantages over the other tools. First, it can classify TEs into no less than nine orders, according to Wicker's classification (versus only four for TEclass and REPCLASS). It performs well for classification into these nine orders, particularly for the dataset for which blast homology searches were not possible, due to the possibility of carrying out HMM profile searches (22% of TEs are well classified and only 16% are misclassified). This is particularly useful when looking at a species phylogenetically distant from the species from which the known TEs were obtained. It performed well for the Repbase-diff dataset, which simulates a standard dataset: 51% of TEs were well classified and 11% were misclassified for classification to order level (for the nine available orders). If we consider only the four REPCLASS levels of classification, PASTEC classified 59% of TEs well and misclassified only 3% of TEs. In the same conditions, REPCLASS classified 67% of TEs correctly and misclassified 11% of TEs (almost four times more than PASTEC).

A second advantage of PASTEC is its output format. We paid particular attention in the development of this tool to the generation of a file readable by humans, containing all the information required for manual curation by a biologist. Furthermore, PASTEC highlights the TEs for which manual curation is particularly necessary, by specifying whether the classification was ambiguous.

Finally, PASTEC's modular architecture makes it possible to add new analyses or decision rules without difficulty. Expert users can access a configuration file, to change diverse parameters.

The main perspective for improving PASTEC concerns classification to the superfamily level, not only through blast searches, but also on the basis of the HMM profiles detected in the TEs. http://urgi.versailles.inra.fr/Tools/REPET

## Supporting Information

Figure S1 **Venn diagram (Repbase-diff dataset) for class I TEs.** The number of well classified class I TEs is shown in brackets. The numbers within the Venn diagram are the numbers of TEs well classified by each tool, with the overlaps indicating those well classified by several tools.
(TIF)

Figure S2 **Venn diagram (Repbase-diff dataset) for class II TEs.** The number of well classified class II TEs is shown in brackets. The numbers within the Venn diagram are the numbers of TEs well classified by each tool, with the overlaps indicating those well classified by several tools.
(TIF)

Figure S3 **Venn diagram (Repbase-diff dataset) for LINE/SINE TEs.** The number of well classified LINE/SINE TEs is shown in brackets. The numbers within the Venn diagram are the numbers of TEs well classified by each tool, with the overlaps indicating those well classified by several tools.
(TIF)

Figure S4 **Venn diagram (Repbase-diff dataset) for helitron TEs.** The number of well classified helitron TEs is shown in brackets. The numbers within the Venn diagram are the numbers of TEs well classified by each tool, with the overlaps indicating those well classified by several tools. Note: TECLASS does not classify helitron TEs to order level.
(TIF)

Figure S5 **Venn diagram (Repbase-diff dataset) for LTR TEs.** The number of well classified LTR TEs is shown in brackets. The numbers within the Venn diagram are the numbers of TEs well classified by each tool, with the overlaps indicating those well classified by several tools.
(TIF)

Figure S6 **Venn diagram (Repbase-diff dataset) for TIR TEs.** The number of well classified TIR TEs is shown in brackets. The numbers within the Venn diagram are the numbers of TEs well classified by each tool, with the overlaps indicating those well classified by several tools. Note: TECLASS does not classify TIR TEs to order level.
(TIF)

Table S1 **An example of PASTEC output.** We present two TEs: the first is classified as a LTR and the second is a potentially chimeric TE (chimera between a helitron and LARD). The output is a tabular file providing the maximum amount of information to facilitate interpretation by biologists.
(DOC)

Table S2 **Sensitivity/specificity for ClassI and ClassII TEs.**
(DOCX)

Table S3 **Sensitivity/specificity for LINE/SINE TEs.**
(DOCX)

Table S4 **Sensitivity/specificity for helitrons TEs.**
(DOCX)

Table S5 **Sensitivity/specificity for LTR TEs.**
(DOCX)

Table S6 **Sensitivity/specificity for TIR TEs.**
(DOCX)
