# Supplementary figures and images for: PASTEC: An Automatic Transposable Element Classification Tool
Source: PLoS One. 2014 May 2;9(5):e91929. doi: 10.1371/journal.pone.0091929 (PMC4008368; doi:10.1371/journal.pone.0091929)

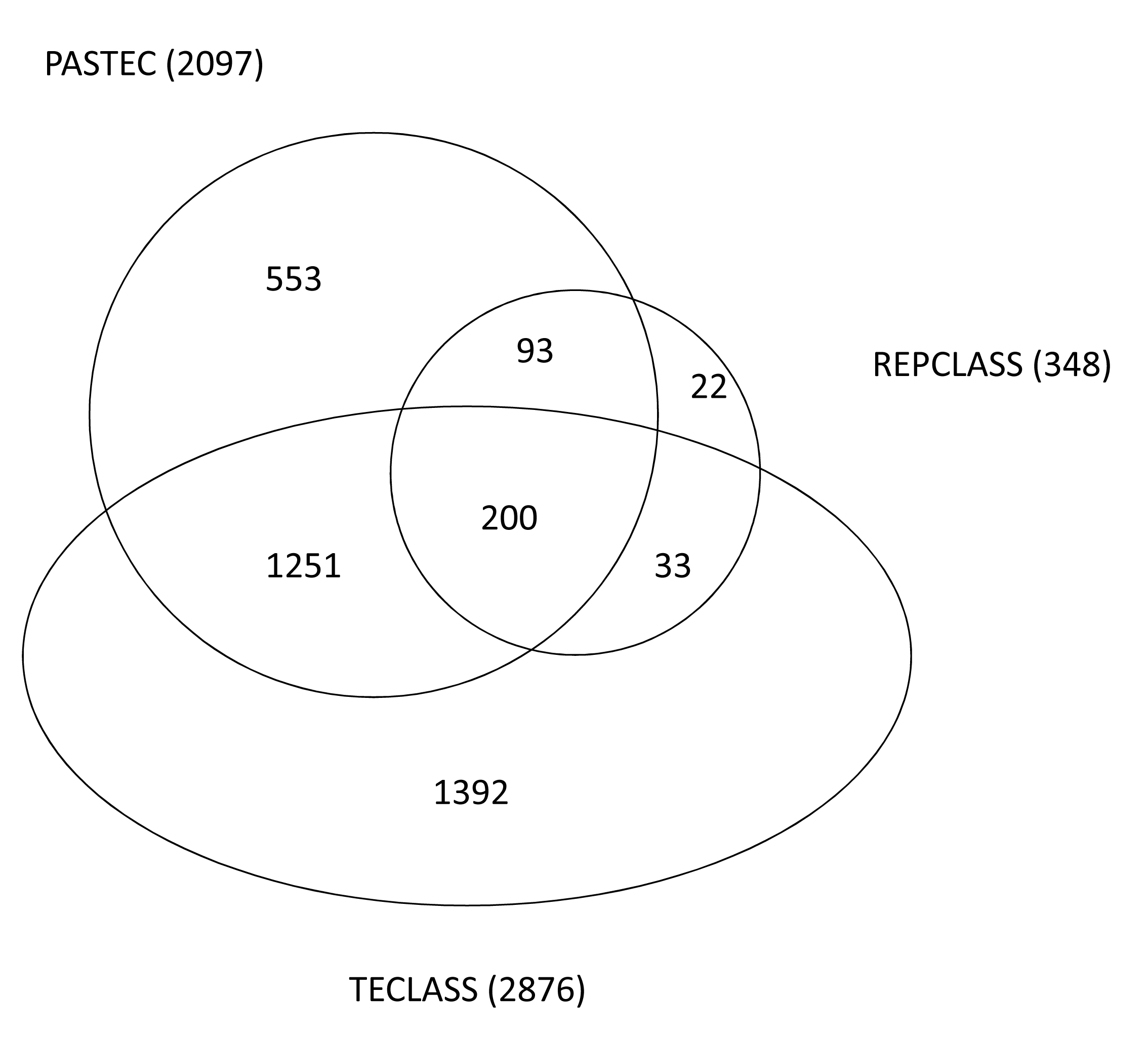

Supplement: Figure S1 — Venn diagram (Repbase-diff dataset) for class I TEs. The number of well classified class I TEs is shown in brackets. The numbers within the Venn diagram are the numbers of TEs well classified by each tool, with the overlaps indicating those well classified by several tools. (TIF) [file pone.0091929.s001.tif]

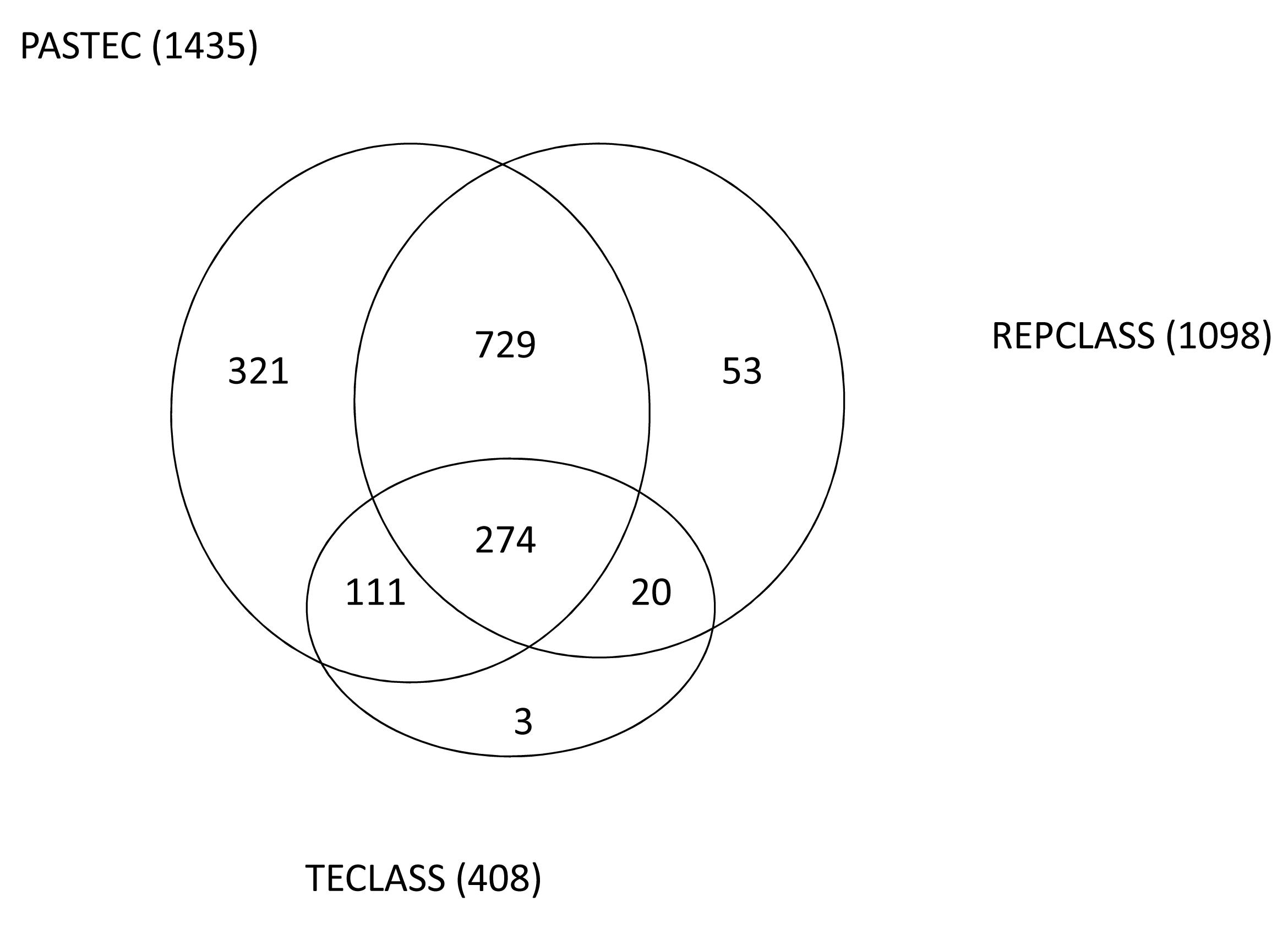

Supplement: Figure S2 — Venn diagram (Repbase-diff dataset) for class II TEs. The number of well classified class II TEs is shown in brackets. The numbers within the Venn diagram are the numbers of TEs well classified by each tool, with the overlaps indicating those well classified by several tools. (TIF) [file pone.0091929.s002.tif]

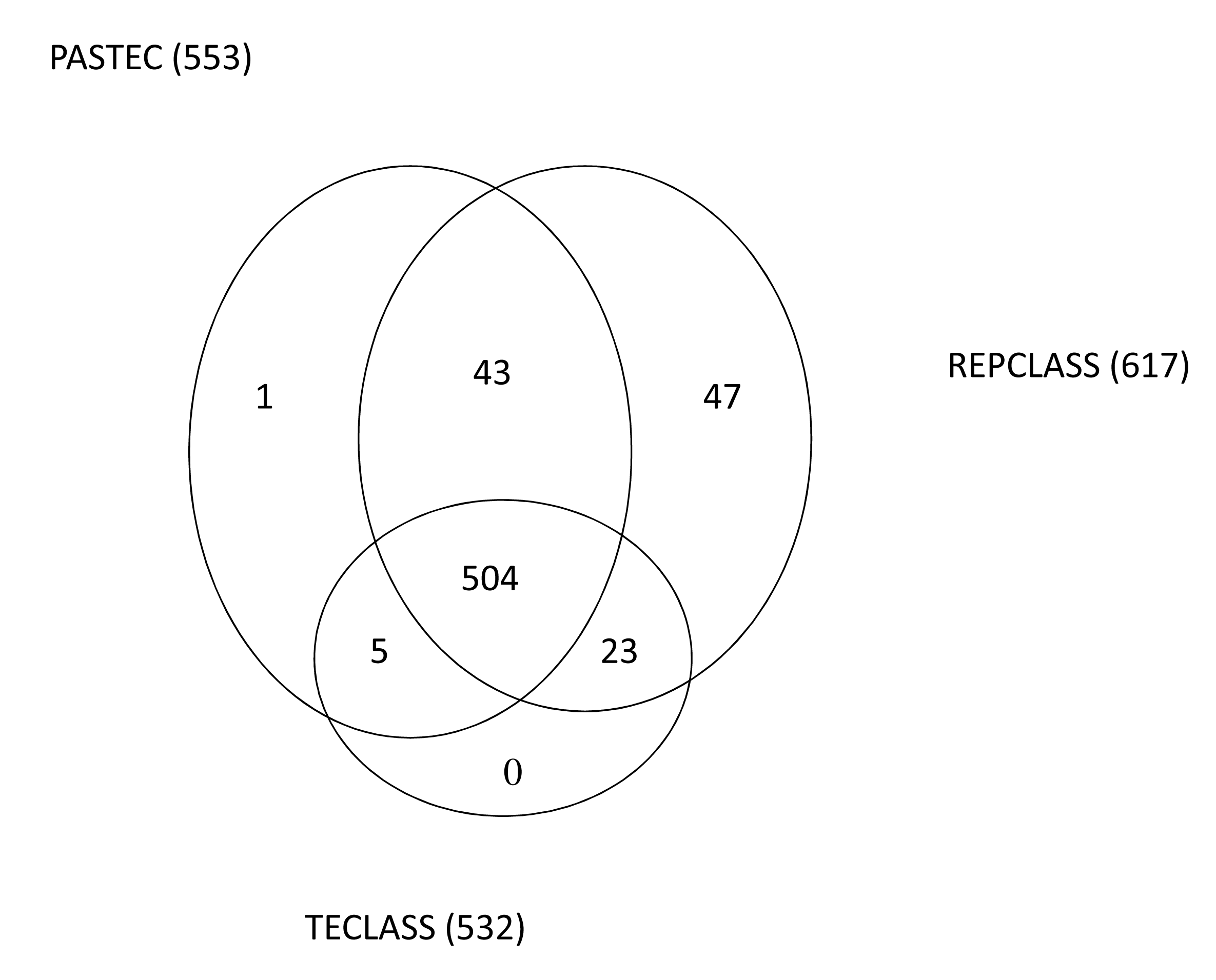

Supplement: Figure S3 — Venn diagram (Repbase-diff dataset) for LINE/SINE TEs. The number of well classified LINE/SINE TEs is shown in brackets. The numbers within the Venn diagram are the numbers of TEs well classified by each tool, with the overlaps indicating those well classified by several tools. (TIF) [file pone.0091929.s003.tif]

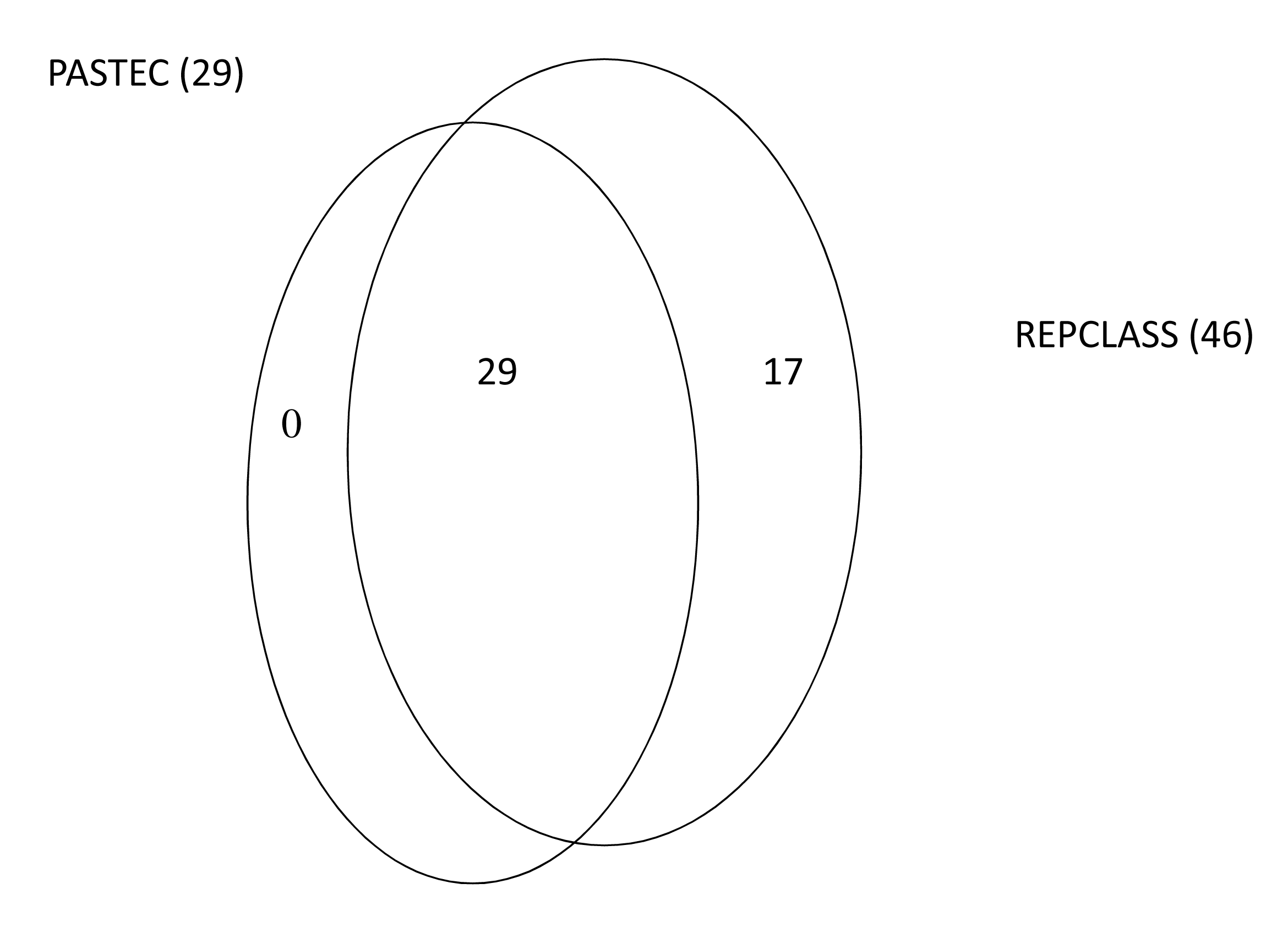

Supplement: Figure S4 — Venn diagram (Repbase-diff dataset) for helitron TEs. The number of well classified helitron TEs is shown in brackets. The numbers within the Venn diagram are the numbers of TEs well classified by each tool, with the overlaps indicating those well classified by several tools. Note: TECLASS does not classify helitron TEs to order level. (TIF) [file pone.0091929.s004.tif]

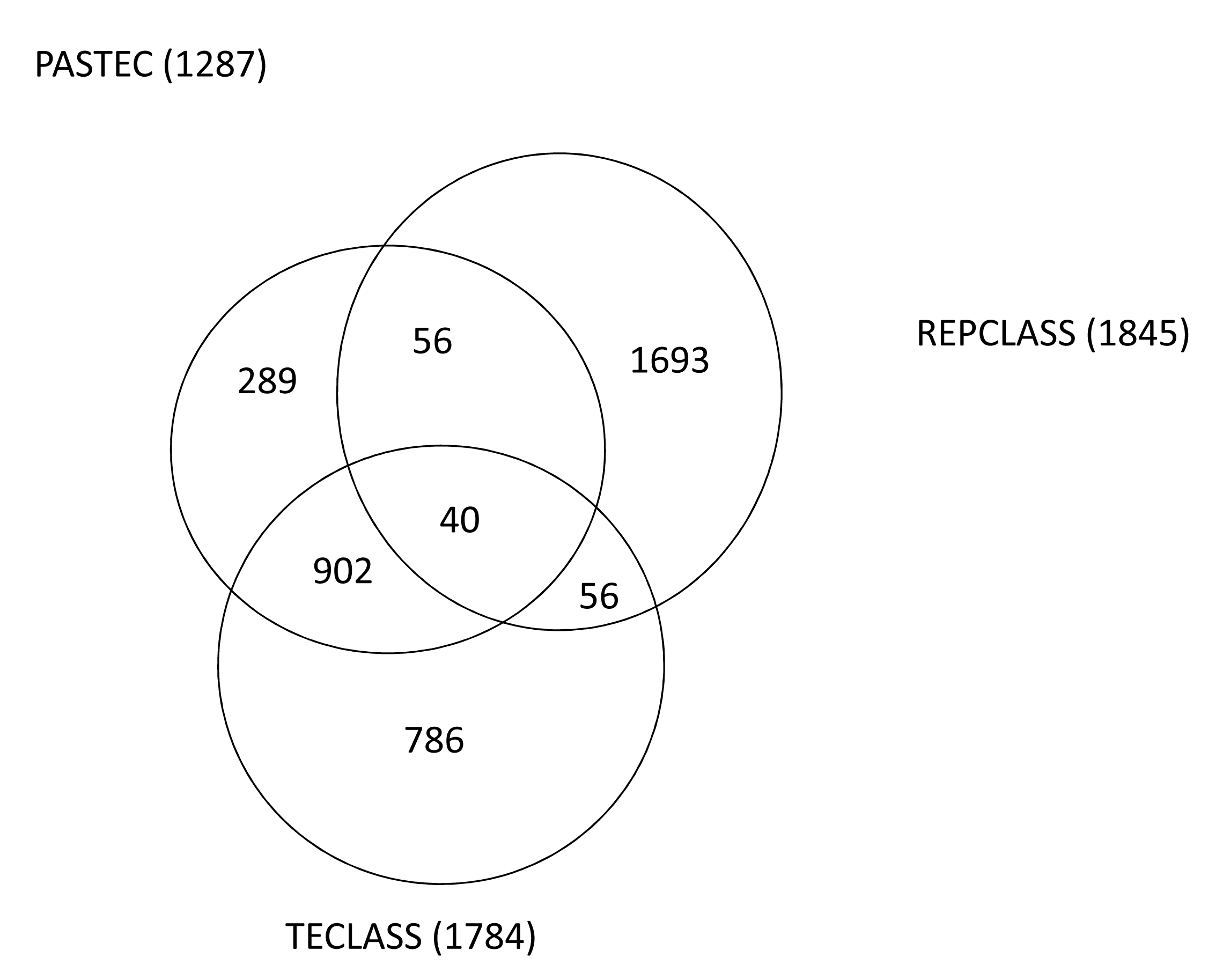

Supplement: Figure S5 — Venn diagram (Repbase-diff dataset) for LTR TEs. The number of well classified LTR TEs is shown in brackets. The numbers within the Venn diagram are the numbers of TEs well classified by each tool, with the overlaps indicating those well classified by several tools. (TIF) [file pone.0091929.s005.tif]

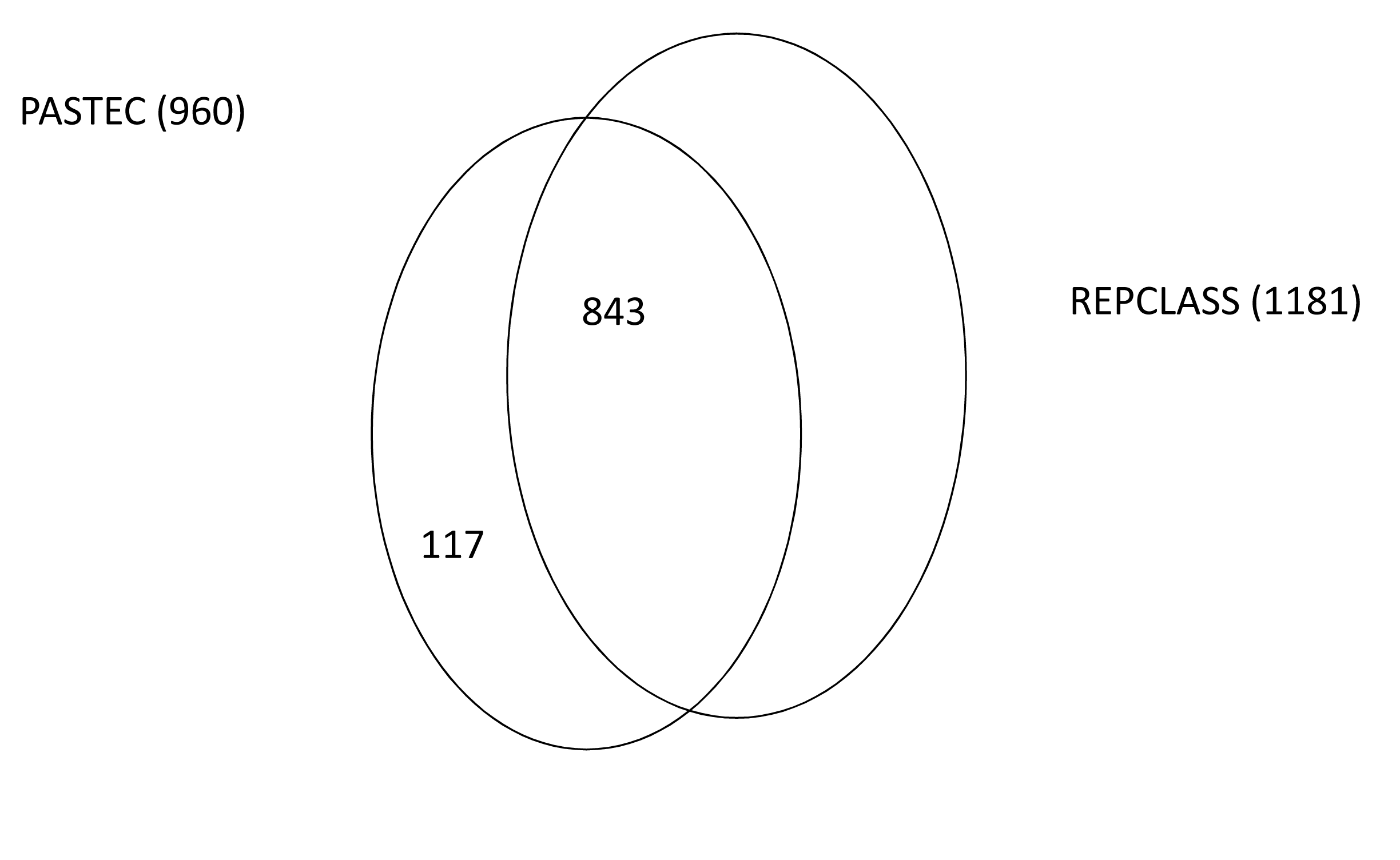

Supplement: Figure S6 — Venn diagram (Repbase-diff dataset) for TIR TEs. The number of well classified TIR TEs is shown in brackets. The numbers within the Venn diagram are the numbers of TEs well classified by each tool, with the overlaps indicating those well classified by several tools. Note: TECLASS does not classify TIR TEs to order level. (TIF) [file pone.0091929.s006.tif]
