# Supplementary material for: PASTEC: An Automatic Transposable Element Classification Tool
Source: PLoS One. 2014 May 2;9(5):e91929. doi: 10.1371/journal.pone.0091929 (PMC4008368; doi:10.1371/journal.pone.0091929)
Supplement: Table S1 — An example of PASTEC output. We present two TEs: the first is classified as a LTR and the second is a potentially chimeric TE (chimera between a helitron and LARD). The output is a tabular file providing the maximum amount of information to facilitate interpretation by biologists. (DOC) [file pone.0091929.s007.doc]

| RLX-comp_fTest14220638-L-B4-Map1 | 8119 | + | ok | I | LTR | complete | CI=71; coding=(TE_BLRtx: STALKER2_I:classI:LTR_retrotransposon: 39.88%, STALKER2_LTR:classI:LTR_retrotransposon: 100.00%, STALKER4_I:classI:LTR_retrotransposon: 57.60%; TE_BLRx: GYPSY11_AG2p:classI:LTR_retrotransposon: 6.38%, GYPSY15_AG2p:classI:LTR_retrotransposon: 6.38%, GYPSY17_AG2p:classI:LTR_retrotransposon: 6.27%, TABOR_DA-I_1p:classI:LTR_retrotransposon: 73.93%, TABOR_DA-I_2p:classI:LTR_retrotransposon: 93.37%; profiles: PF00078.19_RVT_1_RT_35.2: 99.59%, PF00078.19_RVT_1_RT_32.0: 99.59%, PF00077.12_RVP_AP_1.0: 85.71%, PF00665.18_rve_INT_32.0: 94.67%); struct=(TElength: >4000bps; TermRepeats: termLTR: 433; ORF: >1000bps, >3000bps AP RT RT INT); other=(HG_BLRn: FBtr0089123_Dmel_r4.3: 16.55%; SSRCoverage=0.18) |
| --- | --- | --- | --- | --- | --- | --- | --- |
| XXX-chim_fTest14220638-L-B10-Map1 | 3677 | + | PotentialChimeric | noCat | Helitron|LARD | NA | CI=20; coding=(TE_BLRtx: DNAREP1_DM:classII:Helitron: 12.46%, DNAREP1_DYak:classII:Helitron: 40.86%; HG_BLRn: FBtr0089185_Dmel_r4.3: 5.03%); struct=(TermRepeats: termLTR: 381; SSRCoverage=0.08) |
