# Supplementary material for: PASTEC: An Automatic Transposable Element Classification Tool
Source: PLoS One. 2014 May 2;9(5):e91929. doi: 10.1371/journal.pone.0091929 (PMC4008368; doi:10.1371/journal.pone.0091929)
Supplement: Table S2 — Sensitivity/specificity for ClassI and ClassII TEs. (DOCX) [file pone.0091929.s008.docx]

**Table S2**. Sensitivity / specificity for ClassI and ClassII TEs.

|  | Class I | | Class II | |
| --- | --- | --- | --- | --- |
|  | Se (%) | Sp (%) | Se (%) | Sp (%) |
| PASTEC | 53 | 91 | 91 | 53 |
| REPCLASS | 9 | 70 | 70 | 9 |
| TECLASS | 72 | 26 | 26 | 72 |

Note: REPCLASS do not differentiate LINE and SINE at the order Level
