# Supplementary material for: PASTEC: An Automatic Transposable Element Classification Tool
Source: PLoS One. 2014 May 2;9(5):e91929. doi: 10.1371/journal.pone.0091929 (PMC4008368; doi:10.1371/journal.pone.0091929)
Supplement: Table S3 — Sensitivity/specificity for LINE/SINE TEs. (DOCX) [file pone.0091929.s009.docx]

**Table S3** Sensitivity / specificity for LINE/SINE TEs

|  | LINE/SINE |  |
| --- | --- | --- |
|  | Se (%) | Sp (%) |
| PASTEC | 86,8 | 99,7 |
| REPCLASS | 96,4 | 99,4 |
| TECLASS | 11,1 | 85,6 |
