# Supplementary material for: PASTEC: An Automatic Transposable Element Classification Tool
Source: PLoS One. 2014 May 2;9(5):e91929. doi: 10.1371/journal.pone.0091929 (PMC4008368; doi:10.1371/journal.pone.0091929)
Supplement: Table S4 — Sensitivity/specificity for helitrons TEs. (DOCX) [file pone.0091929.s010.docx]

**Table S4**. Sensitivity / specificity for helitrons TEs.

|  | Helitron | |
| --- | --- | --- |
|  | Se (%) | Sp (%) |
| PASTEC | 52,7 | 99,9 |
| REPCLASS | 83,6 | 89,7 |
| TECLASS | NA | NA |

Note: TECLASS do not classified Helitrons TEs at the order level.
