# Supplementary material for: PASTEC: An Automatic Transposable Element Classification Tool
Source: PLoS One. 2014 May 2;9(5):e91929. doi: 10.1371/journal.pone.0091929 (PMC4008368; doi:10.1371/journal.pone.0091929)
Supplement: Table S5 — Sensitivity/specificity for LTR TEs. (DOCX) [file pone.0091929.s011.docx]

**Table S5**. Sensitivity / specificity for LTR TEs.

|  | LTR | |
| --- | --- | --- |
|  | Se (%) | Sp (%) |
| PASTEC | 39,1 | 99,5 |
| REPCLASS | 58,7 | 50,1 |
| TECLASS | 59,4 | 6,5 |
